# Strategies and Challenges of Microbiota Regulation in Baijiu Brewing

**DOI:** 10.3390/foods13121954

**Published:** 2024-06-20

**Authors:** Pengpeng Zhang, Yanbo Liu, Haideng Li, Ming Hui, Chunmei Pan

**Affiliations:** 1 College of Biological Engineering, Henan University of Technology, Zhengzhou 450001, China; ppz0224@163.com (P.Z.); haidengli129@163.com (H.L.); huiming@haut.edu.cn (M.H.); 2 College of Food and Biological Engineering (Liquor College), Henan University of Animal Husbandry and Economy, Zhengzhou 450046, China; yanboliu@hnuahe.edu.cn

**Keywords:** Baijiu, brewing microbiota, functional strains, biofortification, regulatory strategies

## Abstract

The traditional Chinese Baijiu brewing process utilizes natural inoculation and open fermentation. The microbial composition and abundance in the microecology of Baijiu brewing often exhibit unstable characteristics, which directly results in fluctuations in Baijiu quality. The microbiota plays a crucial role in determining the quality of Baijiu. Analyzing the driving effect of technology and raw materials on microorganisms. Elucidating the source of core microorganisms and interactions between microorganisms, and finally utilizing single or multiple microorganisms to regulate and intensify the Baijiu fermentation process is an important way to achieve high efficiency and stability in the production of Baijiu. This paper provides a systematic review of the composition and sources of microbiota at different brewing stages. It also analyzes the relationship between raw materials, brewing processes, and brewing microbiota, as well as the steps involved in the implementation of brewing microbiota regulation strategies. In addition, this paper considers the feasibility of using Baijiu flavor as a guide for Baijiu brewing regulation by synthesizing the microbiota, and the challenges involved. This paper is a guide for flavor regulation and quality assurance of Baijiu and also suggests new research directions for regulatory strategies for other fermented foods.

## 1. Introduction

Fermented food is a kind of food that gains unique flavor by controlling the growth of microorganisms and the action of microbial enzymes to transform the ingredients in the raw materials [1,2]. Baijiu is a traditional fermented beverage in China. It is made using Daqu as a saccharification starter and raw materials such as sorghum, wheat, corn, rice, and glutinous rice. The production process involves natural inoculation, open fermentation, distillation, aging, and blending [3,4,5]. The process of Baijiu brewing is intricate and closely tied to various production conditions, such as the climate of the production site, the environment, brewing water, grain, and Daqu [6]. Therefore, Chinese Baijiu has a diverse range of flavors.

The microbiota plays a vital role in the fermentation process of Baijiu, as it depends on the nutrients in the raw materials to survive. Through its own community succession and growth metabolism, it forms a large number of fermentation metabolites, which lay the foundation for the unique Baijiu body style [4,7,8]. Baijiu is brewed using the natural enrichment and open fermentation strategy [9]. During the brewing process, the microbial system is affected by various production conditions. The microbiota that naturally develops during this process not only collects microorganisms that are conducive to brewing but also those that are harmful to Baijiu brewing, resulting in the production of Baijiu with unstable quality, low yield, high production cost, and so on [10]. Therefore, microbiota, brewing technology, and raw materials are the three key factors that affect the typicality and stability of Baijiu’s flavor.

In general, the quality of Baijiu can be intuitively reflected by its typicality and stability, which are directly related to its flavor [11]. During the brewing process, microbiota dominates the metabolism of various raw material components, leading to the metabolization of most of the flavors of Baijiu [11,12]. Regulating the brewing microbiota has proven to be an effective method for improving the quality of Baijiu. However, a clearer understanding of the brewing technology mechanism is necessary. Currently, production is often based on past experience, which leads to instability in the quality of Baijiu due to a lack of understanding of the brewing mechanism. To stabilize and improve the quality of Baijiu, it is necessary to conduct thorough research on the sources of brewing microorganisms, their interactions, and the impact of raw materials and technology on the final product. Furthermore, the solid-state brewing method of Baijiu prohibits the addition of non-fermentable substances from external sources. To obtain stable and high-quality Baijiu, there are two methods: adding microorganisms to change the initial microbiota, or adjusting the technology and raw materials to influence the brewing microbiota succession. These methods can regulate the production of Baijiu. Currently, scholars are using multiomics technologies such as metagenomics and multivariate statistical methods like source tracker and partial least squares to address the aforementioned issues. This research will pave the way for the gradual realization of regulated and standardized ecological Baijiu brewing [13,14,15].

This paper systematically reviews the origin and composition of microbiota at different brewing stages from the perspective of brewing microbiota. It also discusses the steps involved in implementing microbial intervention in ecological brewing strategies and the relationship and regulation between raw materials, technology, and brewing microecology. This article reflects on the feasibility of regulating the ecological brewing of Baijiu through the synthesis of microbiota oriented to Baijiu flavor. It also discusses the potential challenges involved. The article provides a reference for the flavor regulation and quality control of Baijiu. Additionally, it suggests a new direction for research on regulation strategies for other fermented foods.

## 2. The Source and Composition of the Microbiota for Baijiu Brewing

Many traditional fermented foods and beverages are produced by adding traditional fermentation agents [16], such as Baijiu [17], Japanese sake [18], cheese [19], and so on. However, the microbial enrichment in these traditional fermenters is mainly derived from the feedstock and the environment [20]. For Baijiu, the open fermentation model results in the microbiota during the fermentation period in the Baijiu cellar being composed of two main components, partly from the microorganisms of the Daqu and partly from the environment (air, pit mud, tools, etc.). Environmental changes may cause some instability, but with the gradual standardization and perfection of brewing technology, the succession of microbiota in Baijiu brewing can still be traced. For Baijiu with similar brewing sites or the same flavor, the composition and distribution of the core microbiota driving fermentation are somewhat similar during the various stages of brewing [21,22].

### 2.1. Sources of Microbiota in Baijiu Brewing

The process of traditional Baijiu brewing can be divided into two stages: the fermentation of Daqu and the fermentation of fermented grains [11]. The traditional process uses wheat as the main ingredient, with the addition of a certain amount of peas and barley to produce Daqu [7]. The grains are moistened, crushed, and shaped before being sent to the fermentation chamber. There, they undergo natural inoculation and open fermentation maturation before they are ready for use [23]. Daqu serves as a saccharification fermentation agent in Baijiu brewing and also contributes to the construction of the brewing microbiota, which drives the formation of Baijiu fermentation and flavor styles. Therefore, the enrichment and growth of microorganisms in Daqu are closely related to the brewing of Baijiu.

The raw materials are not sterilized before shaping Daqu. Therefore, during the maturation process, microorganisms from the raw materials and the environment enrich and interact with each other, resulting in a unique and stable microbiota in Daqu [24]. The microbiota of Daqu primarily comprises bacteria and fungi. Previous studies utilized high-throughput sequencing technology along with Source Tracker to analyze the traceability of microorganisms in Daqu. The findings revealed that the fungal community in Daqu mainly originated from workshop tools and floors, including *Saccharomycopsis*, Pichia, Rhizopus, *Sterigmatomyces* and *Aspergillus*. The bacterial community was mainly derived from raw materials (wheat) and included *Gammaproteobacteria*, *Alphaproteobacteria,* and *Bacillus*. In addition, shop tools and floors contribute a portion of the bacterial sources such as *Bacillus* and *Pediococcus*. The air inside and outside the shop brings *Acetobacter*, *Levilactobacillus*, *C. paralimentarius,* and *P. kudriavzevii* to the Daqu [25,26,27].

Due to the unique Baijiu brewing technology and the differences in raw materials and production environments, the treated grains will incorporate many different microbiotas before fermentation in the cellar, such as the Daqu, tools, air, production floor, pit mud, etc. [13,27]. However, research has shown that there are still discernible patterns in the sources of brewing microorganisms for different types of Baijiu. Tools, workshop floors, and air have been identified as the primary sources of bacterial communities, which consist mainly of *Lactobacillus*, *Bacillus*, *Weissella*, and *Kroppenstedtia*. Daqu is the main contributor of fungi during the fermentation of fermented grains, including some aerobic and facultative fungi such as *Thermoascus*, *Thermomyces*, *Pichia*, *Saccharomycopsis,* and *Eurotium*. Pit mud was a major contributor of anaerobic bacteria including *Clostridium*, *Petrimonas*, *Methanobacterium,* and *Sedimentibacter* [13,14,28]. Currently, most of the traceability studies on brewing microbiota are limited to a specific period or genus level of brewing. There are fewer studies on the traceability of microorganisms throughout the fermentation process of Daqu or fermented grains. Research on this subject will not only help us to understand the origin of the brewing microbiota but also identify the sources of beneficial or harmful microorganisms in Baijiu production. This information can be used to adjust and standardize production techniques to ensure the high-quality and healthy development of Baijiu.

### 2.2. Composition of Baijiu Brewing Microbiota

#### 2.2.1. Microbiota Composition of Matured Daqu

The classification of Daqu types mainly uses the highest fermentation temperature as the standard for each category. Daqu is categorized into high-temperature Daqu (60–70 °C), medium–high-temperature Daqu (55–60 °C), medium-temperature Daqu (50–55 °C), and low-temperature Daqu (40–50 °C) [27,29]. The physical and chemical properties, microbial community, and flavor composition of different types of Daqu often have significant differences [30,31]. During the fermentation and maturation process of Daqu, the natural environment and raw materials create the basic microbiota. The microbiota gradually forms a stable community due to the cross-talk of physicochemical properties such as temperature, humidity, and pH, as well as metabolic activities. This community drives multiple activities that result in the formation of flavor and function, including saccharification and esterification.

Daqu fermentation is a complex natural process that involves microbiota. This microbiota plays a crucial role in the fermentation of Baijiu, providing enzymes and flavor substances that contribute to the unique style of Baijiu [32]. During the initial phase of microbial research on Daqu, the composition of Daqu microorganisms was primarily studied using traditional culture-dependent methods, including isolation, purification, and molecular biology identification [10]. However, this method can only provide an understanding of the microorganisms that can be cultured in Daqu. Additionally, it is time-consuming and complicated, and does not allow for a comprehensive understanding of the microbiota of Daqu.

In recent years, microbiomics technologies, such as targeted amplicon sequencing (16S rRNA genes in prokaryotes, 18S rRNA and ITS genes in eukaryotes), metagenomics, metatranscriptomics, and others, have been increasingly used to study brewing microorganisms [1,33,34]. Furthermore, researchers have observed significant differences in microbial community abundance between Daqu samples using 16S rRNA and ITS amplicon sequencing techniques. The highest bacterial diversity was observed in medium–high-temperature Daqu, while the highest fungal diversity was observed in high-temperature Daqu [29]. The microbial community composition varied among different types of Daqu, as shown in Table 1. The marker microorganisms of high-temperature Daqu were mainly composed of *Kroppenstedtia*, *Thermoascus*, *Thermomyces*, *Aspergillus*, and *Bacillus*. The marker microbiota of medium–high-temperature Daqu contains mainly *Weissella*, *Lactobacillus*, *Thermoascus*, *Thermomyces,* and *Aspergillus*. *Weissella*, *Lactobacillus*, *Candida*, *Thermoascus*, and *Leuconostoc* were predominant in the marker microbiome of medium-temperature Daqu. And the low-temperature macrobiotics contain mainly *Saccharomycopsis*, *Lactobacillus*, *Aspergillus*, *Bacillus,* and *Pantoea*. The variations in microbial composition may be attributed to differences in fermentation temperature. Although microbiota can differ among Daqu of the same type, monitoring of microorganisms during fermentation indicates that the microbiota gradually stabilizes and becomes more uniform as fermentation progresses and temperature increases within a certain limit. Therefore, it has become an industry consensus that temperature is a crucial physical and chemical factor in the production process of Daqu. By controlling the process temperature, the quality and output of Daqu can be improved.

#### 2.2.2. Composition of the Microbiota during the Fermentation Stage of Fermented Grains

The Daqu produced at different peak temperatures is used in the brewing of different types of Baijiu after maturation [29]. For example, high-temperature Daqu is mainly used in the production of sauce-flavor Baijiu [47]. Medium–high- and medium-temperature Daqu is often used in the brewing of strong-flavor Baijiu [48]. Low-temperature Daqu is mainly used in the brewing of light-flavor Baijiu [49]. The production of traditional Baijiu involves an important stage known as the fermentation of grains. After crushing, soaking, steaming, and drying the grains with the appropriate amount of water, they are mixed with the full fermentation of Daqu without distillation of the solid. This mixture is called fermented grains [50]. The production of various compounds cannot be separated from the growth and metabolism of microbiota in fermented grains. Therefore, understanding the composition and succession of microbiota in fermented grains is the key to controlling the quality of Baijiu and improving product quality. For fermented grain fermentation, the whole fermentation process is actually a dynamic change from microaerobic fermentation to anaerobic fermentation. The process can be divided into two stages. The first stage is the alcohol production stage, which involves the production of high levels of ethanol. During this stage, aerobic fungi such as molds and yeasts serve as the core functional microorganisms. Once the oxygen is exhausted, fermentation proceeds to the next stage. The second stage is referred to as the advanced acid production stage, during which acetic and lactic acids are produced. The core functional microorganisms in this stage are represented by *Lactobacillus*, a microaerobic or anaerobic bacteria [33,50].

In addition to the various types of Baijiu, there are also differences in the ratios of raw materials, production technology, and brewing environments. Consequently, China has developed 12 types of flavored Baijiu. The four basic flavors of Baijiu are sauce-flavor, strong-flavor, light-flavor, and rice-flavor [51,52]. The essence of Baiju brewing is the process of growth, metabolism, and accumulation of microorganisms in fermented grains. In this paper, we initially analyzed the dominant microbiota in the fermented grains of the four basic flavor types of Baijiu (Table 2). Although the brewing microecology of different types of Baijiu has been constructed by many parties, it is worth noting that a wide range of microorganisms are widely present in the Baijiu brewing process, such as *Lactobacillus*, *Aspergillus*, *Pichia*, *Saccharomyces*, *Rhizopus*, etc. (Table 2). In addition, different types of Baijiu brewing also have their unique signature microorganisms. For example, *Thermoascus*, *Thermomyces*, *Byssochlamys,* and *Kroppenstedtia* are unique to the brewing microecology of sauce-flavor Baijiu (Table 2). This may be related to its special stacking fermentation before cellaring. Stacking fermentation is an open fermentation process (peak stacking temperature of 50 °C, 4–5 days) [53]. During this process, the microbiota in fermented grains is screened under the stress of high temperatures and eventually forms a unique microbiota prior to cellaring [54,55]. This also indicates that brewing technology is an effective way to shape the brewing microbiota. Therefore, exploring the mechanism of the brewing process on brewing microecology and Baijiu body flavor may become a hot spot in future research, which will provide a reference for the high-quality development and stable output of Baijiu.

## 3. Microbiota Regulation of Baijiu Based on Functional Strains of Bacteria

Chinese Baijiu is the world’s largest distilled liquor in terms of production and consumption [73]. It is also one of the top six distilled liquors in the world. Water and ethanol make up 98–99% (*v*/*v*) of the total volume of Baijiu, with only 1–2% (*v*/*v*) comprising the flavor substances that contribute to its unique style. It is important to note that flavor is the most crucial characteristic of Baijiu, and consumer satisfaction and acceptance depend heavily on it [74]. In recent years, advancements in extraction techniques and high-precision trace detection methods (such as GC-IMS and GC × GCTOF-MS) have led to the identification of over 2000 flavor compounds in Baijiu. These compounds primarily consist of esters, alcohols, aldehydes, acids, ketones, nitrogen compounds, aromatic compounds, and terpenoids [75,76,77]. The formation of flavors in Baijiu cannot be separated from the metabolic activities of various microorganisms in the brewing microecology [78,79]. The open fermentation strategy and complex brewing process used in the production of Baijiu have resulted in a very complex composition of brewing microbiota, which has led to the production of Baijiu with unstable quality and low production efficiency. The resolution and regulation of the core brewing microbiota are key to solving the above problems. The composition and succession pattern of core functional microorganisms and their interaction mechanisms are examined during the brewing microecology process. The core microbiota is used as a guide to select and breed functional microbial strains, strengthen brewing ecological fermentation, and achieve top-down regulation of the microbial community. This will help regulate brewing microecology, control the fermentation process, and stabilize Baijiu production.

### 3.1. Microbial Intervention Baijiu Brewing Strategy

Baijiu production involves numerous microorganisms, resulting in a complex microbiota. This complexity poses a significant challenge for the microbiota-based regulation of Baijiu. To regulate Baijiu fermentation, it is essential to ensure positive biofortification for brewing while maintaining normal fermentation and preserving the original flavor profile and mouthfeel.

The identification of core functional microbiota is a prerequisite for the realization of targeted regulation. With the continuous advancement of microbiomics and statistical analysis tools, precise identification of core microbiota from the complex brewing microecology is gradually becoming a reality. The core microbiota is a collection of microorganisms that can maintain a certain amount of biomass during the fermentation stage, and at the same time are closely related to the production of flavor substances, the physicochemical properties of carriers, and other microorganisms [7,11,39]. The core microbiota is defined based on several conditions and requires the use of statistical tools such as Pearson/Spearman correlation coefficients, microbial covariance network analysis, and partial least squares regression. Researchers have developed a core microbiota discrimination strategy that combines multivariate omics analysis (metatranscriptomics, metagenomics, metabolomics, etc.), physical and chemical property analysis, and multidimensional correlation statistical tools. Some researchers have made this strategy more specific. In the first step, GC-MS, GC-TOF/MS, GC-TOF/MS, and metabolomics techniques were used to describe the key flavor compositions of Baijiu while focusing on the pattern of physicochemical factor evolution during fermentation. In the second step, the microbial composition of the brewing ecology was scanned using microbiomics (metatranscriptomics, metagenomics, etc.). Finally, correlation analysis between multiple data (microbiota, flavor, physicochemical properties) was performed using a multidimensional correlation analysis tool, and the core microbiota was displayed through a visualization tool [11]. Under the guidance of this strategy, the core microbiota of various natural fermentation systems, such as Daqu, vinegar, yellow wine, and tea, was successfully identified [33,34,80,81].

**Figure 1:**
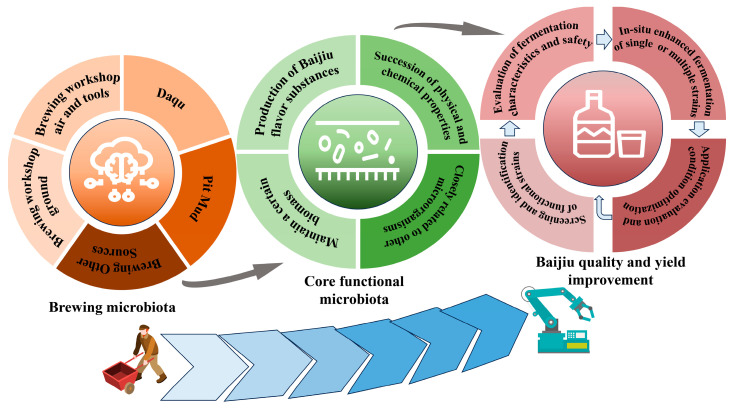
Steps in the implementation of the biofortification strategy for Baijiu.

Biofortification involves isolating and screening functional strains using microbial isolation technology, guided by the core functional microbiota composition and traceability analysis. In situ fermentation is then applied with single or multiple strains after fermentation characterization and safety evaluation to achieve high-quality and efficient production of Baijiu [10,82,83]. Figure 1 displays the implementation of the biofortification strategy. To begin, it is necessary to identify the microbial origin of Daqu, fermented grains, or pit mud using microbiomics techniques. This will lay the foundation for the subsequent identification of harmful strains and screening of functional strains. Next, the core microbial discrimination strategy was utilized to distinguish the core microorganisms from the harmful ones in Baijiu brewing. Microbial isolation techniques and directed culture histology were used to screen and characterize functional strains based on the obtained results [84,85]. The strains were evaluated and applied to the in situ fermentation system of Daqu or Baijiu to enhance fermentation. The fermentation conditions were then optimized by adjusting the inoculation method and the amount of functional microorganisms to achieve optimal performance of the strains. This strategy can help distinguish between functional and harmful strains more effectively and accurately. In production, harmful stray bacteria can be reduced through standardized practices, while fermentation microbiota can be regulated through biological enhancement. This allows for the gradual realization of the controllable, efficient, and mechanized production of Baijiu.

### 3.2. Single Strain Biological Enhanced Baijiu Brewing

Biofortification of Baijiu microecology with functional strains can optimize the microbial community structure, flavor metabolism, and yield output of the fermentation system [71,86]. The effects of biofortification on Baijiu fermentation are many and varied. There are also many ways to implement biofortification. Under the guidance of microbial traceability and core microbiota strategy, the sources of functional microbial strains are more abundant. Various approaches can be used to enhance functional strains, including producing fortified Daqu, pure-strain Daqu (bran Daqu), and strain suspensions. It is important to note that the selection of different strains for strengthening the application should be based on their own functional characteristics, production technology, top-level design, and economic practicality, among other factors. Single-microorganisms-fortified fermentation is one of the important biofortification methods. Yeast, bacteria, and molds are the three major components in the microecology of Baijiu brewing, which are directly related to the fermentation of Baijiu and the production of brewing flavors [10,87,88]. Currently, isolation and screening of strains for functionality basically revolves around flavor and function.

Yeast is closely related to ester flavor and ethanol production in Baijiu brewing [83,89,90]. This was also demonstrated by the yeast deficiency fermentation experiments of Du et al. [91]. In order to improve the yield and quality of Baijiu, high production of esters has been used as a requirement for the screening of target yeast strains. After obtaining the target strains, the researchers increased the content of esters such as ethyl hexanoate and ethyl acetate in Baijiu by making fortified Daqu or pure-strain suspensions [38,71,87]. It has also been shown that the addition of a single exogenous yeast strain also increases the levels of compounds such as ethanol, phenylethanol, and tetramethylpyrazine in the fermentation system [83]. *Wickerhamomyces anomalus* increases esters such as ethyl caproate and ethyl acetate in fermented grains while decreasing the content of higher alcohols when added to the solid-state fermentation system of Baijiu [92].

Bacteria, as an important part of the microbiota during Baijiu brewing, not only provide Baijiu with abundant flavor material components but also provide different types of enzymes such as proteases, amylases, esterases, etc., to drive fermentation [10]. Among them, *Bacillus spp*. are widely present in the production process of Baijiu and have high diversity, so *Bacillus spp*. have been widely investigated. Analysis of the application of *Bacillus subtilis* to fermented Daqu revealed that the addition of exogenous *Bacillus subtilis* promoted the growth of *Aspergillus*, *Thermomyces,* and *Rasamsonia* strains in Daqu [88]. In addition, the researchers also inoculated *Bacillus licheniformis* into Daqu using different inoculation methods to investigate the differences between the enhancement effects of these methods on Daqu fermentation. The results showed that different inoculation methods had significant effects on the physicochemical, microbial diversity, and volatile flavor components of Daqu [93]. The addition of functional strains produced certain bioturbation effects on the fermentation system and enhanced the characteristics of the fermentation system. The inclusion of endogenous *Bacillus spp.* in Daqu not only perturbed the in situ microbiota of Daqu but also significantly enhanced the liquefaction and saccharification power of Daqu [94]. Bacteria other than *Bacillus* are also participants in biofortification. *Caproicibacterium lactatifermentans* is the main producer of caproic acid in high-quality pit mud. In order to cultivate a high-quality and stable fortified pit mud, *Caproicibacterium lactatifermentans* was added to the pit mud and cultivated, and the fortified pit mud produced an abundance of fatty acids and ethyl esters [95].

In Baijiu brewing, the molds in the microbiota mainly originate from the production environment; *Aspergillus oryzae*, *Aspergillus oryzae* and *Rhizopus oryzae* are the main brewing molds. They can provide liquefaction, saccharification, and proteolysis for fermentation, and are the main power source for the degradation of macromolecules such as starch and proteins during the fermentation process, providing flavor substances or their precursors for fermentation [96,97]. However, there are few studies on which mold strains enhance Baijiu brewing. Potential influencing factors may be that the strong proliferative properties of molds have an inhibitory effect on other fungi in the microecology, and may disrupt the in situ ecological balance after artificial enhancement, affecting fermentation [98,99]. After the mold-enhanced fermentation, the fermentation system will have a stronger saccharification force, resulting in the rapid accumulation of reducing sugars and other precursors. This can disrupt the balance between saccharification and alcohol production in the Baijiu brewing system, ultimately affecting normal fermentation [100,101].

### 3.3. Multi-Strain Biological Enhanced Baijiu Brewing

The production of traditional Chinese Baijiu involves a co-fermentation system with multiple microorganisms [102,103]. Enhanced fermentation of a single strain can cause a larger bioturbation effect on the microbiota of Baijiu brewing. This effect can significantly increase the abundance of specific strains while inhibiting the growth of certain core microorganisms. To further regulate the microbiota of Baijiu brewing and improve Baijiu quality, it is important to consider this impact. A variety of bacterial strains were used together to enhance Baijiu fermentation. The results showed that the multi-strain enhancement did not significantly disturb the in situ microbiota of brewing and could improve Baijiu quality [82,104]. The ratio of strains when combining different types of strains for fortification is the key to the efficacy of fortification. The combination of *Bacillus velezensis* and *Bacillus subtilis* promoted the growth succession of several bacteria (Bacillus, Lactobacillus) in Daqu and enhanced the liquefaction, esterification, and saccharification of Daqu (*p* < 0.05) while significantly increasing pyrazine, ester, and alcohol volatile compounds (*p* < 0.05) [105]. Combinations of *Bacillus subtilis*, *Staphylococcus epidermidis*, and *Millerozyma farinosa* were used to fortify Daqu at different fungal-to-bacterial ratios of 1:0.2, 1:0.5, and 1:1. The optimized ratios were found to significantly increase the esters and aromatic compounds of Daqu, promote saccharification and fermentation, and positively regulate the microbial community succession of Daqu [106]. *Bacillus velezensis* and *Bacillus subtilis* were combined in a 1:1 ratio to fortify Daqu. After comparison, it was found that the multi-strain fortification had a positive effect on the microbiota and metabolome of Daqu, and promoted saccharification, esterification, and fermentation [107]. The above suggests that optimization of the ratios is a critical step towards the enhanced application of multiple strains and is essential for positive regulation and quality improvement of the fermentation.

There are many other methods of biofortification outside of fortified Daqu, such as pure-strain suspensions and multi-strain bran Daqu. The direct participation of *Clostridium butyricum*, *Rummeliibacillus suwonensis,* and *Issatchenkia orientalis* in the fermented grains in the same concentration of bacterial suspension at a ratio of 1:1:1 resulted in a significant increase in flavor compounds such as ethyl butyrate in the fermented grains (*p* < 0.05), and the alcohol content was also increased, which ultimately enhanced the quality of the product [108]. *Aspergillus niger*, *Aspergillus erythropolis*, *Saccharomyces cerevisiae,* and ester-producing yeast are optimized to promote the saccharification and fermentation of Baijiu by optimizing the combination of flavor-forming bran Daqu and simulating fermentation, which enriches the microbiota of brewing and enhances the content of various Baijiu flavoring substances [109]. A variety of functional microorganisms through the optimization of single-factor experiments and orthogonal experiments to optimize ratio can strengthen the quality of Baijiu; the production of Baijiu with cellar incense, five grain incense and other composite flavor, Baijiu style of mellow and full-bodied, pure, sweet and refreshing, with the Sichuan style of light-flavor Baijiu typical style to achieve the standard of superior Baijiu [110].

## 4. Microbiota Regulation of Baijiu Based on Technology and Raw Materials

**Figure 2:**
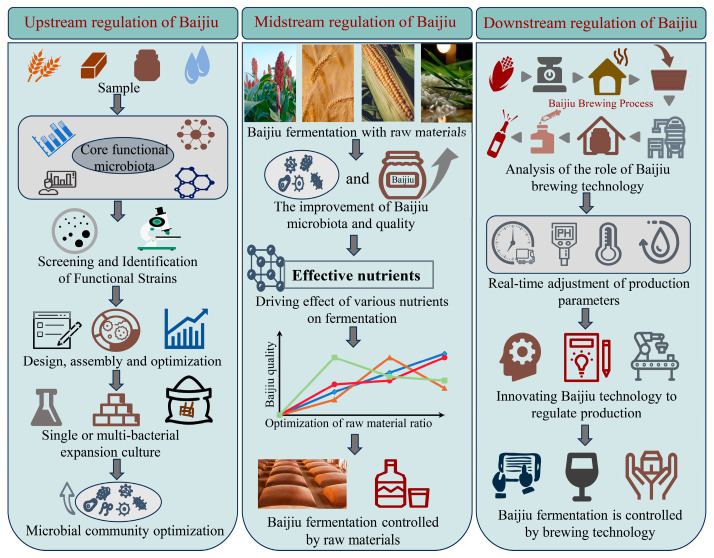
The implementation of coupling multiple modulation techniques.

Although bio-enhanced fermentation strategies can promote fermentation and regulate the microbiota of Baijiu, the transformation from traditional Baijiu brewing to controllable, mechanized, and efficient modern Baijiu brewing has not yet been achieved. Raw materials and brewing technology are important components in shaping the microbiota of Baijiu brewing. Raw materials and brewing technology can regulate the growth and metabolic activities of microorganisms by controlling the nutrient diversity and environmental, physical, and chemical factors of the brewing microbiota. Therefore, a biofortification strategy was employed upstream of brewing to enhance the fermentation process. The midstream brewing process optimizes the ratios of raw materials to regulate the growth and metabolism of the brewing microbiota. In the downstream brewing process, control is utilized to promote co-survival and co-fermentation among brewing microorganisms (Figure 2). Multiple control instruments are combined to achieve better control effects and promote the controlled evolution of Baijiu brewing.

### 4.1. Relationship between Raw Materials and Microbiota and Regulatory Strategies

Microbiota as a tool to drive fermentation and flavor generation in spontaneously fermented foods will produce different flavors based on different raw materials [111]. Unlike some distilled liquors made from a single grain (such as whiskey and vodka), Baijiu is made from a variety of grains, including sorghum, wheat, rice, corn, and barley. The production of multiple raw materials makes Baijiu rich in nutrients, promotes microbial growth and metabolism, and gives Baijiu a stereoscopic texture and multiple flavors. Therefore, raw materials are one of the key factors in determining and regulating the quality of fermented foods.

The flavor composition of Baijiu brewing is directly affected by the choice of grain types and ratios used as raw materials. It is important to carefully select these factors to achieve the desired flavor profile. There are differences in the flavor of Baijiu made from different mono-grains. Sorghum has the advantage of producing a superior Baijiu [112]. There is a correlation between sorghum varieties with different traits and Baijiu quality. Specifically, Japonica rice sorghum and glutinous rice sorghum have differences in physicochemical properties and nutrient contents. By comparing the Baijiu made from the two different trait sorghums, significant differences were found in the ester and alcohol contents of the resulting Baijiu. The primary flavor component of Baijiu made from japonica rice sorghum is ethyl acetate, while the primary flavor component of Baijiu made from glutinous rice sorghum is ethyl lactate [113]. Under the same conditions, barley, purple wheat, and pure wheat were each used to produce high-temperature Daqu. A comparison of their microbial community analyses revealed that different raw materials had a significant impact on the formation of Daqu’s marker microbiota [114]. *Hovenia acerba* is a tall tree that is rich in polysaccharides, amino acids, flavonoids, and other components. It has been used in recent years in Baijiu brewing as a food–medicine plant [115,116,117]. The addition of *Hovenia acerba* increased the content of flavor substances, including organic acids and polyphenols, as well as the abundance and diversity of microbial communities in Baijiu. This resulted in an enhancement of the quality of Baijiu [118].

To investigate the response mechanism of microbial growth and metabolism, as well as flavor material output and raw materials in Baijiu fermentation, a study utilized different varieties of barley rice (Heilaoya and Dulihuang) for Baijiu brewing. The microbiota, physicochemical properties, and flavor production during Baijiu fermentation were monitored. The study found that variations in the sugar spectrum of raw materials were a significant factor in the quality differences of Baijiu. The growth and composition of the microbiota were mainly influenced by fructose and glucose [119]. Specific sugars significantly affect the microbiota in the fermentation system. Furthermore, the content and composition of other effective nutrients, such as amino acids, also impact the fermentation process. High-throughput sequencing and metatranscriptomics were utilized to analyze Baijiu fermentation batches with varying amino acid contents. The study found that differences in amino acid content affect fungal succession in the fermentation system. Specifically, serine promotes the growth of *Zygosaccharomyces* and ethanol production, while amino acids can promote the production of ethanol and volatile flavor compounds by influencing carbohydrate and amino acid metabolism [15]. Currently, the effects of various nutrients found in raw brewing materials on brewing microecology are not yet fully understood. Further research is needed to better understand the response interactions of brewing microbiota with raw brewing materials. Based on this information, producers can adjust the ratio of raw grains in real time to regulate the quality output of Baijiu, resulting in improved efficiency and stability while maintaining or enhancing quality.

### 4.2. Relationship between Technology and Microbiota and Regulation Strategies

The microbiota involved in Baijiu brewing undergoes continuous screening and domestication, resulting in the development of highly functional and adaptable characteristics [11]. Quality instability in Baijiu production may have multiple causes. One possible cause is the introduction of harmful strains during the brewing process. Another possible cause is the insufficient analysis of traditional Baijiu brewing technology, which may result in the inability to adjust the technology parameters in time to cope with changing production conditions. To investigate the impact of traditional brewing techniques on microbiota, researchers have studied the mechanisms behind various traditional brewing methods.

Stacking fermentation is a traditional Baijiu brewing technology that enhances the brewing microbiota through enrichment and blooming. It serves as an intermediate operation between Daqu and cellar fermentation and has been widely applied in Baijiu brewing. However, its role in driving the enrichment of volatile flavor and microorganisms in Baijiu is not yet fully understood. To investigate the mechanism, researchers have examined the stacking process of different types of Baijiu. The findings indicate that pH and moisture are crucial environmental factors for the formation of microbiota during the stacking process. Implementing the stacking process promotes the production of alcohols, esters, and other compounds, as well as the growth and enrichment of bacteria [54,55,120]. Prolonged fermentation in the cellar is a traditional brewing technology for strong-flavor Baijiu. It typically lasts for 100–220 days and significantly affects the metabolic succession of brewing microorganisms and the production of flavor substances [121]. The physicochemical factors that significantly affect fermentation are temperature, alcohol content, and acidity. Additionally, the accumulation of time favors the improvement of flavor and Baijiu quality [62]. To enhance the utilization of raw brewing materials, Baijiu is fermented twice using the same batch of raw materials. This traditional brewing technology results in two different Baijiu outputs. Research has shown that the initial fermentation process has a greater fungal diversity, while the subsequent fermentation process is richer in microorganisms and bacteria. The starch content is the primary physicochemical factor contributing to this difference, resulting in distinct flavor profiles of fermented grains during various fermentation cycles [65]. During the stacked fermentation period, sauce-flavored Baijiu utilizes pelleting to increase the temperature. A study of the fermented grains before and after pelleting showed that turning the grains redistributed water and acidity, leading to improved flavor production and increased microbial abundance [122]. Research on traditional Baijiu brewing technology and its inheritance can help to better understand its intrinsic mechanism of action. This understanding can then be used to regulate the quality and output of Baijiu through real-time adjustment of parameters during actual production.

However, some new production issues cannot be resolved by adjusting the parameters of the traditional brewing process. Therefore, it is necessary to update the process based on the traditional process or new technological innovations to regulate actual production. During the solid-state fermentation of Baijiu, a significant amount of brownish-yellow liquid is produced at the bottom of the fermented grains, also known as yellow water. This liquid is a by-product of Baijiu production and is rich in esters, alcohols, and microorganisms [123,124]. In Baijiu production, yellow water is typically discarded due to the high cost of treatment. To reduce production costs, a new environmentally friendly Baijiu production process has been proposed. This process involves pretreating rice hulls (auxiliary brewing materials) with yellow water, which softens the rice hulls, enhances their elasticity, and improves the efficiency of solid-state fermentation [125]. Distillers’ grains are a by-product of Baijiu distillation, containing rich crude fiber, crude protein, and other nutrients. Discarding them would cause a serious waste of resources and environmental pollution. To utilize distillers’ grains efficiently, one can create low-cost activated carbon by heating and steam carbonization processes. This method can effectively solve the problem of turbidity in low-alcohol Baijiu while retaining its flavor substances [126]. The birth of these innovative technologies will lead to the green, efficient, and healthy development of Baijiu brewing.

## 5. Microbiota Regulation of Baijiu Brewing Based on Synthetic Microbiota

**Figure 3:**
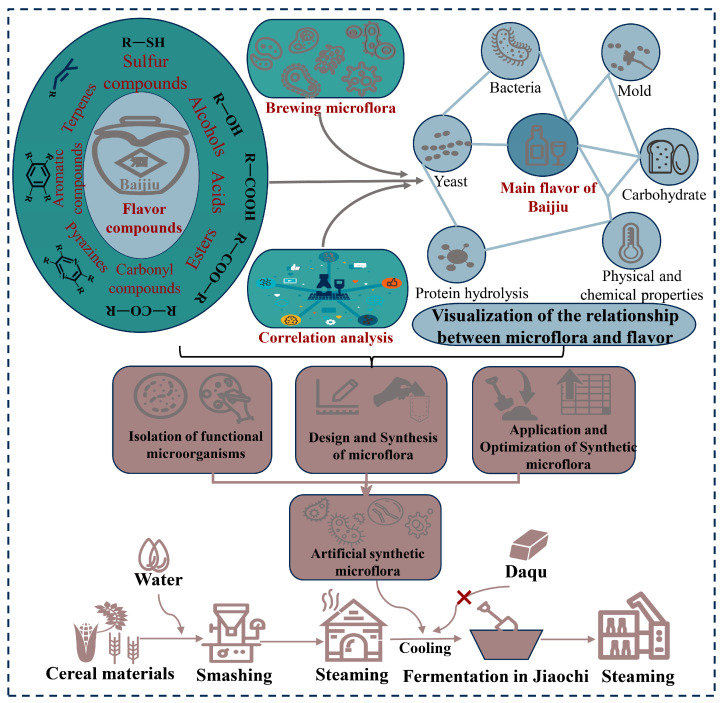
Strategies and applications of artificially synthesized microbiota.

Consumers’ preferences for fermented foods, such as Baijiu, are closely related to the composition of the food’s flavor substances. Compounds associated with the senses of smell and taste are known as flavor substances [127]. In the Baijiu fermentation micro-ecological system, the microbial population converts various substrates to produce flavor substances. The microorganisms have a clear division of labor, with some being responsible for producing hydrolytic enzymes that catalyze the hydrolysis of carbohydrates and proteins, while others are responsible for producing catalytic enzymes that catalyze the metabolism of various flavor compounds. To regulate and improve the quality of fermented foods, natural fermentations are transformed into controlled fermentations using synthetic microbiota. This is achieved by understanding the link between microbiota and the formation of flavor compounds. Flavors can guide microbial isolation, assembly, and deployment to create artificial colonies that replace complex natural fermentation patterns (Figure 3) [128].

However, unlike traditional fermented foods such as wine, bread, and pickles, Baijiu has a wide variety of flavor substances that contribute to the formation of its unique taste. In fact, there are over 300 different flavor compounds that contribute to its distinct style [77]. The fermentation of Baijiu is a highly complex process that involves raw materials, technology, and environmental factors. Interpreting the mechanism of Baijiu brewing has been challenging due to the complexity of these factors. Even controlled fermentation of Baijiu was considered unattainable in the past. In recent years, microbial isolation and analysis technology has rapidly developed, leading to efforts towards the controlled fermentation of Baijiu. The idea of flavor-oriented controlled fermentation through the synthesis of microbiota is gradually becoming possible. In order to analyze the coexistence relationship between the production of flavor compounds and microorganisms in Baijiu brewing, an artificial simplified microbiota containing five core microorganisms, including *Lactobacillus*, *Saccharomyces*, *Pichia*, *Geotrichum,* and *Candida*, was constructed and used as a substitute for Daqu in brewing, and the results showed that this artificial microbiota achieved 77.27% flavor compound mimicry, and the fermentation process showed dynamic characteristics similar to natural fermentation [129]. The microbiota present in the pit mud plays a direct role in producing various volatile acids and alcohols during the brewing process of concentrated Baijiu [51]. To construct a simplified microbiota of the pit mud with high capric acid production, a top-down strategy was employed. Its use in Baijiu production promotes the production of esters such as ethyl caproate and ethyl valerate, and the bacteria do not produce odor compounds [130]. A total of twenty key flavor compounds of Baijiu were identified through research. The producers of these key flavor compounds were then distinguished, and a simplified artificial microbiota was constructed using mathematical models to achieve targeted synthetic regulation of multiple key flavors [131].

In summary, the flavor-oriented strategy of constructing synthetic microbiota to regulate the quality output of Baijiu can effectively improve the quality and stability of Baijiu. Currently, the full identification of key Baijiu flavors and their abundance remains incomplete. Additionally, the composition of the core microbiota and the mechanisms of microbial interactions in Baijiu microecology are not yet fully understood. Furthermore, existing microbial isolation techniques are insufficient to achieve the complete isolation of core microorganisms. The application of synthetic microbial fermentation in Baijiu is a slow process due to various challenges. An in-depth understanding of the mechanism of Baijiu brewing is still necessary.

## 6. Conclusions and Prospects

To address the issue of fluctuating Baijiu quality and achieve efficient and standardized production, regulation and management of the brewing process are necessary. Baijiu production involves multi-microbial co-fermentation, making microorganisms essential to the final product. Regulating the quality of Baijiu through microorganisms has been proven to be an effective solution. The first step is to understand the mechanisms behind microbial-driven and -regulated Baijiu fermentation. The second step is to investigate the core microbial sources and interactions between microorganisms. To achieve this, microbial isolation and analysis techniques will be used to modify and improve Baijiu fermentation using single or multiple strains. Previous studies have shown that this approach is effective. However, biotechnology currently has constraints that limit the effectiveness of strain-based bio-enhanced regulation. Therefore, it is necessary to analyze the effects of process and raw materials on brewing microecology and provide assistance accordingly. One approach is to focus on flavor and use multiomics technology to identify the core flavor substances and microorganisms. To ensure consistent brewing, a synthetic microbiota is designed based on different liquor body styles. This stabilizes and modernizes Baijiu production while minimizing the impact of varying brewing environments.

## Figures and Tables

**Table 1:** Marker microbiota of different types of Daqu and study methods.

Types of Daqu	Research Methods	Marker Microbiota Composition	References
High-temperature Daqu	Ultra-high-depth macrogenomic sequencing	*Lactobacillus*, *Weissella*, *Kroppenstedtia*, *Sphingomonas*, *Saccharopolyspora*, *Pediococcus*, *Leuconostoc*, *Staphylococcus*	[35]
	High-throughput sequencing technology (16S rRNA, ITS)	*Kroppenstedtia*, *Bacillus*, *Saccharopolyspora*, *Thermoascus*, *Thermomyces*	[36]
	High-throughput sequencing technology (16S rRNA, ITS)	*Oceanobacillus*, *Thermomyces*, *Kroppenstedtia*, *Thermoascus*	[37]
	ITS High throughput sequencing	*Ascomycota*, *Basidiomycota*, *Thermomyces*, *Thermoascus*, *Aspergillus*	[37]
	Metagenomic sequencing	*Kroppenstedtia*, *Thermoactinomyces*, *Bacillus*, *Acinetobacter*, *Aspergillus*, *Byssochlamy*, *Thermoascus*, *Thermomyces*	[38]
Medium–high-temperature Daqu	High-throughput sequencing technology	*Bacillus*, *Weissella*, *Thermoactinomyces*, *Lactobacillus*, *Thermoascus*, *Thermomyces*, *Kodamaea*, *Aspergillus*	[23]
	Ultra-high-depth macrogenomic sequencing	*Lactobacillus*, *Weissella*, *Kroppenstedtia*, *Sphingomonas*, *Alcelaphine*	[35]
	Metatranscriptomic	*Aspergillus*, *Bacillus*, *Leuconostoc*, *Pediococcus*, *Eurotiales*, *Bacillales*, *Saccharomycetales*	[39]
	High-throughput sequencing technology (16S rRNA, ITS)	*Weissella*, *Thermoascus*, *Bacillus*, *Pichia*, *Thermomyces*, *Lactobacillus*	[40]
Medium-temperature Daqu	High-throughput sequencing technology	*Weissella*, *Lactobacillus*, *Pediococcus*, *Saccharopolyspora*, *Thermoascus*, *Issatchenkia*, *Candida*	[41]
	High-throughput sequencing technology	*Weissella*, *Leuconostoc*, *Bacillus*, *Lactobacillus*, *Thermoascus*, *Thermomyces*, *Candida*	[42]
Low-temperature Daqu	High-throughput sequencing technology	*Lactobacillus*, *Pantoea*, *Staphylococcus*, *Saccharomycopsis*, *Wickerhamomyces*, *Aspergillus*, *Millerozyma*	[43]
	High-throughput sequencing technology	*Kroppenstedtia*, *Bacillus*, *Saccharomycopsis*, *Issatchenkia*, *Cladosporium*, *Aspergillus*	[44]
	Metagenomic	*Bacillus*, *Streptomyces*, *Pantoea*, *Kosakonia*, *Lactiplantibacillus*, *Saccharopolyspora*	[45]
	High-throughput sequencing technology	*Lactobacillus*, *Weissella*, *Pichia*, *Saccharomycopsis*	[46]

**Table 2:** Core microbiota of fermented grains of basic types of Baijiu.

Type of Baijiu	Core Microbiota Composition	References
Sauce-flavor Baijiu/Moutai-flavor Baijiu	*Pichia*, *Schizosaccharomyces*, *Thermomyces*, *Thermoascus*, *Aspergillus*, *Byssochlamys*, *Saccharomyces*, *Lactobacillus*, *Kroppenstedtia, Oceanobacillus*	[56,57,58,59,60]
Luzhou-flavor Baijiu/strong-flavor Baijiu	*Lactobacillus*, *Weissella*, *Acetobacter*, *Aspergillus*, *Caproiciproducens*, *Issatchenkia*, *Pichia*, *Candida*, *Rhizopus*, *Kazachstani*	[61,62,63,64]
Fen-flavor Baijiu/light-flavor Baijiu	*Lactobacillus*, *Pichia*, *Saccharomyces*, *Bacillus*, *Lichtheimia*, *Pantoea*, *Cladosporium*, *Pediococcus*, *Monascus*, *Aspergillus*, *Rhizopus*	[65,66,67,68,69]
Rice-flavor Baijiu	*Rhizopus*, *Saccharomyces*, *Parasitella*, *Absidia*, *Lichtheimia*, *Bacillus*, *Lactobacillus*, *Weissella*, *Pediococcus, Lactococcus*, *Acetobacter*	[70,71,72]

## Data Availability

The original contributions presented in the study are included in the article, further inquiries can be directed to the corresponding author.
